# Direct Comparison of Elastography Endoscopic Ultrasound Fine-Needle Aspiration and B-Mode Endoscopic Ultrasound Fine-Needle Aspiration in Diagnosing Solid Pancreatic Lesions

**DOI:** 10.3390/ijerph19031302

**Published:** 2022-01-24

**Authors:** Marcel Gheorghiu, Zeno Sparchez, Ioana Rusu, Sorana D. Bolboacă, Radu Seicean, Cristina Pojoga, Andrada Seicean

**Affiliations:** 1 Department of Gastroenterology, Iuliu Hațieganu University of Medicine and Pharmacy, 400192 Cluj-Napoca, Romania; marcel.gheorghiu@gmail.com (M.G.); zsparchez@gmail.com (Z.S.); ioana.russu@yahoo.com (I.R.); andradaseicean@gmail.com (A.S.); 2 Department of Gastroenterology, Regional Institute of Gastroenterology and Hepatology “Prof. Dr. Octavian Fodor”, 400192 Cluj-Napoca, Romania; cristinapojoga@yahoo.com; 3 Department of Medical Informatics and Biostatistics, Iuliu Hațieganu University of Medicine and Pharmacy Cluj-Napoca, 400349 Cluj-Napoca, Romania; 4 First Surgical Department, Iuliu Hațieganu University of Medicine and Pharmacy Cluj-Napoca, 400005 Cluj-Napoca, Romania; rseicean@yahoo.com; 5 Clinical Psychology and Psychotherapy Department, Babeș-Bolyai University, 400015 Cluj-Napoca, Romania

**Keywords:** elastography, EUS-FNA (endoscopic ultrasound fine-needle aspiration), endoscopic ultrasound (EUS), fine needle aspiration (FNA), pancreatic cancer, diagnostic, histology

## Abstract

Elastography endoscopic ultrasound (E-EUS) has been proved to be a valuable supplement to endoscopic ultrasound fine-needle aspiration (EUS-FNA) in differentiating solid pancreatic lesions, but the improvement of EUS-FNA guided during E-EUS has not been proven. Our study aimed to evaluate whether E-EUS fine-needle aspiration (E-EUS-FNA) was superior to B-mode EUS-FNA for the diagnosis of solid pancreatic masses and whether the diagnostic rate was affected by specific factors. Our prospective study was conducted between 2019–2020 by recruiting patients with solid pancreatic masses. E-EUS examination was followed by one pass of E-EUS-FNA towards the blue part of the lesion and a second pass of EUS-FNA. The final diagnosis was based on surgery, E-EUS-FNA or EUS-FNA results, or a 12-month follow-up. Sixty patients with solid pancreatic lesions were evaluated. The sensitivity, specificity, and accuracy for diagnosing malignancy using E-EUS-FNA and EUS-FNA were 89.5%, 100%, 90%, 93%, 100%, and 93.3%, respectively, but the differences were not significant. Neither mass location nor the lesion size influenced the results. The lengths of the core obtained during E-EUS-FNA and EUS-FNA were similar. E-EUS-FNA in solid pancreatic lesions was not superior to B-mode EUS-FNA.

## 1. Introduction

The endoscopic ultrasound (EUS) tools for assessing solid pancreatic lesions include strain elastography, which evaluates the stiffness of the tissue based on elastic pattern, strain ratio, or strain histogram. The elastography pattern is green in soft tissues and blue in hard tissues, representing a qualitative assessment [1]. Elastography EUS (E-EUS) assessment proved useful in discriminating solid pancreatic tumors smaller than 30 mm, as showed by a meta-analysis, with a sensitivity of 98% (95% CI: 0.96–0.99, CI = confidence interval), and a specificity of 63% (95% CI: 0.58–0.69), but without the possibility to obtain the tissue acquisition such as EUS fine-needle aspiration (EUS-FNA) or EUS fine needle biopsy (FNB) [2]. Even in the case of lesions smaller than 15 mm, E-EUS sensitivity for malignancy was 84%, with 67% specificity, but with a negative predictive value of 98%, sustaining that a small soft lesion rules out the diagnosis of malignancy [3]. The contrast-enhanced EUS can orientate the needle’s direction inside the tumors to avoid necrosis or vessels, and their combination increases the diagnosis up to 94% [4]. Elastography-guided (EUS) FNA was reported in one retrospective study showing an accuracy of 94.4% [5]. Still, no direct comparison between standard EUS-FNA and elastography EUS-FNA (E-EUS-FNA) was performed [5].

Our study aimed to evaluate if E-EUS-FNA is superior to standard EUS-FNA in solid pancreatic masses diagnosis and identify the factors affecting the diagnostic rate.

## 2. Materials and Methods

### 2.1. Study Design

A prospective study was conducted between May 2019 and October 2020 at the Regional Institute of Gastroenterology and Hepatology Cluj-Napoca, Romania. The hospital institutional review board approved the study (7070/2019), and each participating patient had given written informed consent according to Helsinki guidelines.

### 2.2. Subjects and Data Collection

Patients older than 18 years of age with a computed tomography (CT) diagnosis of solid pancreatic masses requiring a EUS-guided sampling were eligible for the study.

Exclusion criteria were: (1) participant refusal or contraindication to the proposed intervention; (2) a platelet level <50,000/mcL (microlitres) and INR (international normalized ratio) >1.5; (3) prior curative surgical treatment or chemoradiotherapy; (4) a pancreatic tumor with a cystic component; (5) duodenal stenosis impeding the complete EUS assessment; (6) and presence of a biliary metallic or plastic stent.

### 2.3. Study Outcomes and Definitions

The primary outcome of the study was to assess the diagnostic value of E-EUS-FNA compared to EUS-FNA. The second outcome was to find any factors that could influence the diagnostic results for each group.

The eligible patients who agreed to participate in the study were analyzed by EUS, elastography, one pass of E-EUS-FNA, and one pass of EUS-FNA consecutively. As the EUS-FNA could modify the color pattern of elastography, we performed the first pass as E-EUS-FNA and the second pass as B-mode EUS-FNA. A third B-mode EUS-FNA pass was performed and considered for the final diagnosis when a puncture failed or the visual core was <4 mm [6]. Only the endoscopist matched the bottle number with the type of FNA. A second puncture was made one month after the index EUS-FNA whenever the histological analysis was inconclusive.

EUS-FNA, E-EUS-FNA, repeat EUS-FNA, or post-surgical histopathological examination were used for the final diagnosis. A 12-month follow-up was conducted whenever a patient had a negative FNA. The patients were clinically examined at follow-up and received a CT scan and a transabdominal ultrasound at 3-month intervals.

### 2.4. Procedures

A therapeutic linear array echoendoscope (GF-UCT 180 AL5; Olympus, Tokyo, Japan) with an Aloka Prosound F75 ultrasound machine equipped with elastography software was used. All interventions were undertaken by two endoscopists (A.S. and C.P.) using 22G needles (Expect^TM^; Boston Scientific Corporation, Natick, MA, USA). Patients were in the left lateral decubitus position under light sedation (intravenous midazolam). After the EUS assessment, elastography was performed. The region of interest during elastography covered over 50% of the solid lesion and avoided surrounding regions with a very low density, such as vessels or ducts. Elastography patterns can be classified into five types, based on elasticity scores, color patterns, and heterogenicity of distribution of elasticity [1]. The blue color indicated hard lesions (e.g., adenocarcinoma, neuroendocrine tumors) and the green pattern suggested inflammatory lesions.

The first pass of E-EUS-FNA was performed using fanning and the slow-pull technique: the needle was moved to-and-fro ten times within the lesion while an assistant simultaneously slowly pulled the stylet. As described previously, the needle was advanced into the blue part of the targeted lesion, while the green parts of the lesions, suggestive of inflammation, were avoided [4]. Then, the elastography button was switched off and EUS-FNA was performed in the same way as E-EUS-FNA.

### 2.5. Preparation of Samples

The cytopathologist was not present at sample collection. The cores from EUS-FNA and E-EUS-FNA were expelled by re-introduction of the stylets into 10% buffered formalin. The blinded tissue cores (no information regarding the type of pass) were sent for histological analysis. Only the endoscopist knew the match of the type of FNA.

The specimens were measured and paraffin-embedded, stained with hematoxylin-eosin-safran, with or without immunohistochemistry sections. Two experienced pathologists (I.R. and D.R., each had assessed >1000 pancreatic EUS-FNA) blinded to the sample provenance (EUS-FNA or E-EUS-FNA) but with access to the clinical and imaging information independently analyzed the samples.

A coherent tissue sample was considered adequate for histologic examination. Positive specimens, including neuroendocrine tumors (NETs), were classified as malignanct. Specimens with inadequate material or atypia were classified as negative for malignancy (intention-to-diagnose analysis).

### 2.6. Statistical Analysis

Data were summarized as a number (%) whenever qualitative. Mean ± standard deviation was reported for quantitative data that proved to follow a normal distribution; in opposite cases, data were reported as median [Q1 to Q3], where Q is the quartile. The analysis was conducted on an intention-to-diagnose basis [7]. A clinical utility index (CUI) calculator [8] was used to retrieve the metrics of diagnostic performances, and the results were reported with 95% confidence intervals (Wald method). Differences between performance metrics were tested using the Z test for proportions. The applied statistical tests were two-sided, with α = 0.05. The normal distribution of numerical data and the ROC analysis were performed with Statistica (v13.5, StatSoft, Tulsa, OK, USA).

## 3. Results

### 3.1. Patients’ Characteristics

Sixty-five patients were potentially eligible participants; five were excluded after the EUS assessment due to the cystic component that modified the E-EUS evaluation. Sixty patients were evaluated by EUS-FNA and E-EUS-FNA and were included in the analysis. No adverse events were registered in our cohort.

The patients’ characteristics are listed in Table 1. The size of tumors located in the head/uncinate process/isthmus or body/tail was 31.5 ± 7.7 mm and 30 ± 7.9 mm (*p* = 0.498), respectively.

EUS-FNA or E-EUS-FNA gave the final diagnosis in 56 patients, confirmed by surgery in 13 patients, repeated EUS-FNA, and surgery in one case. Negative EUS-FNA and E-EUS-FNA together with follow-up proved benign lesions in three patients.

### 3.2. B-Mode EUS Assessment

Fifty-seven patients had a hypoechoic lesion on EUS, and five of them had high vascularity on a Doppler assessment. Two lesions were isoechoic, and one was hyperechoic and had high vascularity.

### 3.3. E-EUS Assessment

Thirty-seven lesions (61.7%) showed blue homogenous aspects: 34 adenocarcinomas, 1 lymph node, and 2 NETs. Twenty-two lesions showed a blue inhomogeneous aspect: 18 cases of adenocarcinoma, 1 case of schwannoma, 1 renal metastasis, 1 autoimmune pancreatitis, and 1 solid serous cystadenoma. One lesion, a pancreatic clear cell carcinoma metastasis, had a mixt blue-green aspect. The blue homogenous or heterogenous aspect was predictive for adenocarcinoma with 100% (all lesions without any exceptions) sensitivity, 87.5% (64.6 to 100) specificity, 98.1% (94.5 to 100%) positive predictive value, 100% negative predictive value, 0.981 (0.949 to 1.000) positive clinical utility index (CUI+) (excellent for case finding) and 0.85 (0.722 to 1.000) negative clinical utility index (CUI−) (excellent for screening).

The median strain ratio was 24.5 (16.75 to 32.00) for patients with adenocarcinoma. No significant area under the curve (AUC) was observed as to strain ratio for diagnosing adenocarcinoma (AUC = 0.456 (0.215 to 0.696), *p* = 0.688).

### 3.4. The Diagnostic Yield in E-EUS-FNA Compared to EUS-FNA

**Figure 1:**
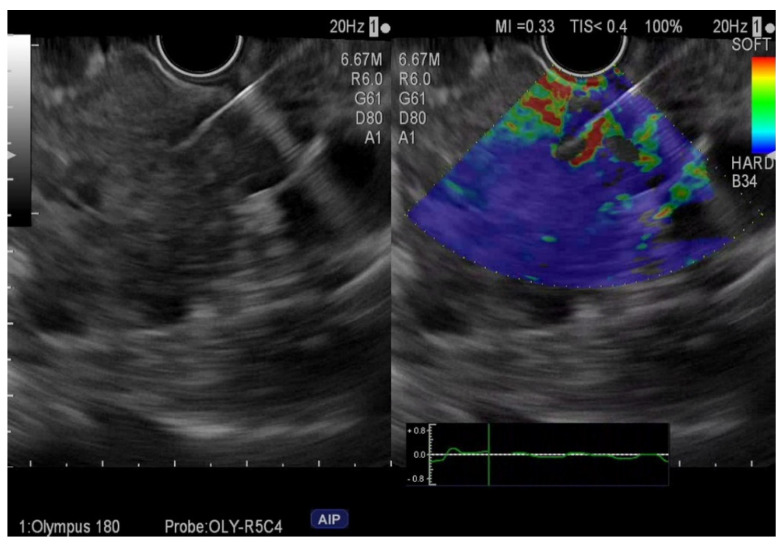
Elastography endoscopic ultrasound fine-needle aspiration of pancreatic adenocarcinoma.

**Figure 2:**
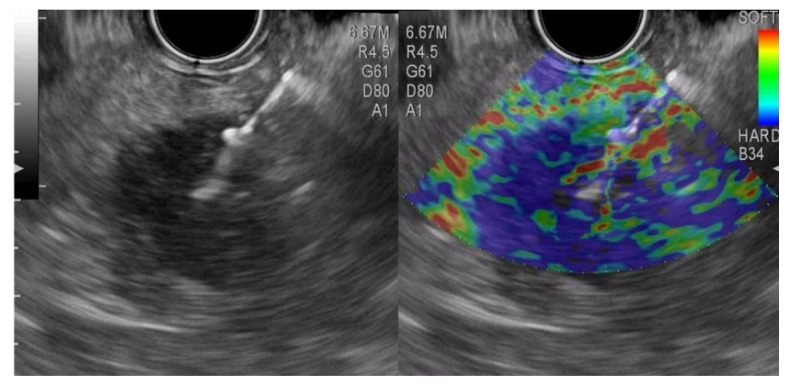
Elastography endoscopic ultrasound fine-needle aspiration from a pancreatic neuroendocrine tumor.

A malignant (primary or metastatic) pancreatic tumor was present in fifty-seven patients. EUS-FNA and E-EUS-FNA exhibit comparable diagnostic performances (Table 2) with excellent performances for case-finding. The E-EUS-FNA false-negative cases were represented by two cases of adenocarcinomas considered as pancreatic intraepithelial neoplasia (PanIn) and four adenocarcinomas. The EUS-FNA false-negative cases were four adenocarcinomas interpreted as PanIn in two cases and two cases negative for malignancy (Figure 1 and Figure 2).

### 3.5. Factors Influencing E-EUS-FNA and EUS-FNA Results

Seven cases had small tumors ≤20 mm (one chronic pancreatitis, one metastasis, and five adenocarcinomas), but size did not influence E-EUS-FNA (*p* = 0.798) or EUS-FNA (*p* = 0.599) diagnostic results.

The location of the lesion in the head of the pancreas did not influence the true positive and true negative results in E-EUS-FNA group samples (*p* = 0.898) or EUS-FNA group samples (*p* = 0.198).

The length of the core obtained in E-EUS-FNA was 24.1 ± 11.5 mm for head lesions and 20.6 ± 10.6 mm for body/tail lesions (*p* = 0.289). The length of the core obtained in EUS-FNA was 23.8 ± 10.1 mm for head lesions and 17.8 mm ± 10.2 mm for body/tail lesions (*p* = 0.048). The length of the core in the E-EUS-FNA group was 22.6 ± 10.3 mm in malignant lesions and 26.7 ± 15.8 mm in benign lesions (*p* = 0.321). The length of the core in EUS-FNA was 22.0 ± 10.5 mm in malignant lesions and 23.6 ± 9.9 mm in benign lesions (*p* = 0.706).

## 4. Discussion

Our study is the first one that proves that guidance during strain elastography of EUS-FNA gives similar diagnostic results as B-mode EUS-FNA. When the pass needle was guided in the blue part of the lesion, the sensitivity was 89.5% compared to 93% during the FNA from the B-mode image, without significant difference, proving the excellent clinical utility in diagnosing malignancy for both methods (Table 2). However, the clinical utility index for screening of malignancy was fair for E-EUS-FNA and moderate for EUS-FNA (Table 2). Color pattern assessment by elastography for diagnosing malignancy was associated with high sensitivity (99%), but with moderate specificity (76%) [9], caused by the rigid behavior of neuroendocrine tumors and some nodules of chronic pancreatitis, which can appear as blue [9]. The semi-quantitative analysis of elastography images using the strain response or hue histogram [9,10,11] did not obtain a better diagnostic rate, concluding that tissue acquisition cannot be replaced by elastography.

Four meta-analyses have shown that EUS-FNA has 85–92% sensitivity, and 96–98% specificity [12,13,14,15] and efforts have been made to increase the diagnostic rate by using complementary tools, such as contrast enhancement or elastography, or by replacing FNA with FNB needles.

A direct comparison between elastography EUS, B-mode EUS, and EUS-FNA proved diagnostic accuracies for pancreatic lesions of 73%, 87%, and 85%, raising the question about the supplemental benefit of E-EUS over conventional B-mode EUS [16]. However, in 50 cases of negative EUS-FNA, E-EUS was associated with 97% sensitivity and 77% specificity, sustaining the role of this procedure in such a subset of patients [17]. Moreover, the combination of both EUS elastography and contrast-enhanced EUS increased the diagnostic sensitivity, specificity, and accuracy to 98.57%, 81.48%, and 93.81%, respectively [18].

Another practical use of elastography is guiding EUS-FNA for sampling pancreatic tumors to precisely target the hardest part of the mass, considering the region with higher suspicion of malignancy. The technique was described in a small descriptive study on 13 patients [19]. Later on, a retrospective study was published on 54 patients with a 25 G needle inserted in the most suspicious part of the lesion under E-EUS with two-needle passes of the needle. They reported a 93.4% sensitivity and 100% specificity, but no control group was present [5]. Compared to our study, where we used only 22 G needles, they used 25 G FNA needles and both studies proved accuracies over 92%. In fact, the size of the needle is not important, as demonstrated in one meta-analysis, which showed no difference for diagnostic accuracy when different size FNA needles were used [20]. FNA in pancreatic masses seems less accurate to FNB in terms of diagnostic rate and adequacy [21], but our study included only patients with EUS-FNA.

Contrast-guided EUS-FNA in sampling pancreatic tumors was previously performed in two randomized controlled trials, one with cross-over assessment with EUS-FNA and the other with parallel groups of EUS-FNA/FNB. The results showed non-inferiority of standard EUS-FNA/FNB [22,23]. The conclusion was that the assessment of tumors is very well performed with the modern EUS platform and that the role of contrast EUS-FNA/FNB should be in case of indeterminate lesions on chronic pancreatitis, infiltrative lesions, or tumors in the context of acute pancreatitis [24], but these situations cannot be applied in the case of E-EUS-FNA.

One major problem when using the qualitative assessment of strain elastography is its variability and subjectivity compared to contrast, so the color to orientate the needle inside the lesion is often changing. Moreover, the pressure produced by advancing the needle during E-EUS-FNA modifies the color pattern, so the real-time needle movement in the blue part of the tumor relies on the initial image. This could explain why the lengths of the core obtained during EUS-FNA were significantly greater than those obtained during E-EUS-FNA and the screening value for malignancy (CUI-) was significantly higher in the case of EUS-FNA.

The controlled arm of B-mode EUS-FNA represents the strength of this study. The limitations of this study are the small number of patients, which could lead to some lack of power. Another limitation is related to the use of core histology exclusively, without cytology assessment. Moreover, the adequacy of samples was based on visual measurement of the core, and we encountered no case of an inadequate sample, but we had several cases of false-negative results in both groups. Another limitation of the study was the absence of randomization. We preferred to have the first pass performed under elastography because it could modify the color pattern of the pancreatic mass, and to have the second pass as B-mode EUS-FNA.

## 5. Conclusions

Our study proved that E-EUS FNA has similar usefulness as B-mode EUS-FNA in the needle guidance for histologic samples of pancreatic lesions. Elastography remains helpful as an imaging tool for orientating towards malignancy diagnosis, but not for guiding FNA. Better results are expected from its association with tissue acquisition using fine needle biopsies.

## Figures and Tables

**Table 1:** The characteristics of assessed patients with solid pancreatic lesions.

Characteristic	Value
Age (years), mean ± SD	66.4 ± 10.04
Male sex, *n* (%)	26 (43.33)
Mass location, *n* (%)	
Head/uncinate process/isthmus	44 (73.33)
Body/tail	16 (26.66)
Mass size (cm), median (Q1 to Q3)	30.00 (29.5 to 35)
Stage	
T1	4 (6.67)
T2	3 (5.00)
T3	35 (58.33)
T4	18 (30.00)
Final diagnosis, *n* (%)	
Adenocarcinoma	52 (86.67)
Neuroendocrine tumors	2 (3.33)
Pancreatic metastases	2 (3.33)
Schwannomas	1 (1.67)
Benign lesions	3 (5)

SD = standard deviation; *n* = number of cases; Q1 = 25th percentile; Q3 = 75th percentile.

**Table 2:** Accuracy metrics for diagnosing malignancy by E-EUS-FNA and EUS-FNA.

Metric	E-EUS-FNA	EUS-FNA	*p*-Value
True positives, *n*	51	53	
True negatives, *n*	3	3	
False negatives, *n*	6	4	
False positives, *n*	0	0	
Sensitivity, % (95% CI)	89.5 (81.5 to 97.4)	93.0 (86.4 to 99.6)	0.4975
Specificity, %	100	100	n/a
Positive predictive value, %	100	100	n/a
Negative predictive value, % (95% CI)	33.3 (2.5 to 64.1)	42.9 (6.2 to 79.5)	0.2789
Accuracy, % (95% CI)	90.0 (82.1 to 97.6)	93.3 (87.0 to 99.7)	0.5135
Negative likelihood	0.11 (0.05 to 0.22)	0.07 (0.03 to 0.18)	0.4439
+CUI	0.895 (0.822 to 0.968)	0.930 (0.870 to 0.989)	0.4975
−CUI	0.333 (0.041 to 0.742)	0.626 (0.116 to 0.742)	0.0013

CUI: clinical utility index, E-EUS-FNA: elastography endoscopic ultrasound fine-needle aspiration, EUS-FNA: endoscopic ultrasound fine-needle aspiration.

## Data Availability

Data is contained within the article.
